# Protocol for isolation of signet ring cells from human gastric mucosa

**DOI:** 10.1016/j.xpro.2023.102695

**Published:** 2023-11-04

**Authors:** Sarah G. Samaranayake, Lauren A. Gamble, Cassidy Bowden, Benjamin L. Green, Amber F. Gallanis, Dilara Akbulut, Niharika Shah, Jonathan M. Hernandez, Jeremy L. Davis

**Affiliations:** 1 Surgical Oncology Program, National Cancer Institute, National Institutes of Health, Bethesda, MD 20892, USA; 2 Laboratory of Pathology, National Cancer Institute, National Institutes of Health, Bethesda, MD 20892, USA

**Keywords:** Cell Biology, Cancer, Molecular Biology

## Abstract

More than 90% of individuals with germline pathogenic CDH1 variants will harbor occult, microscopic foci of signet ring cell carcinomas capable of progressing to advanced diffuse-type gastric cancer. Here, we present a protocol for high viability suspension of signet ring cells from human gastric tissue. We describe the steps for gastric mucosa isolation and tissue dissociation. We then detail procedures for embedding cells into HistoGel for immunohistochemistry staining and additional applications such as flow cytometry and single-cell sequencing.

## Highlights

• Protocol for isolation and dissolution of human gastric mucosa
• Dissociation of mucosa into single-cell suspension with high viability
• Isolated cells suitable for flow cytometry and scRNA-seq applications

## Abstract (editor highlights)

Publisher’s note: Undertaking any experimental protocol requires adherence to local institutional guidelines for laboratory safety and ethics.

## Graphical abstract

**Figure:**
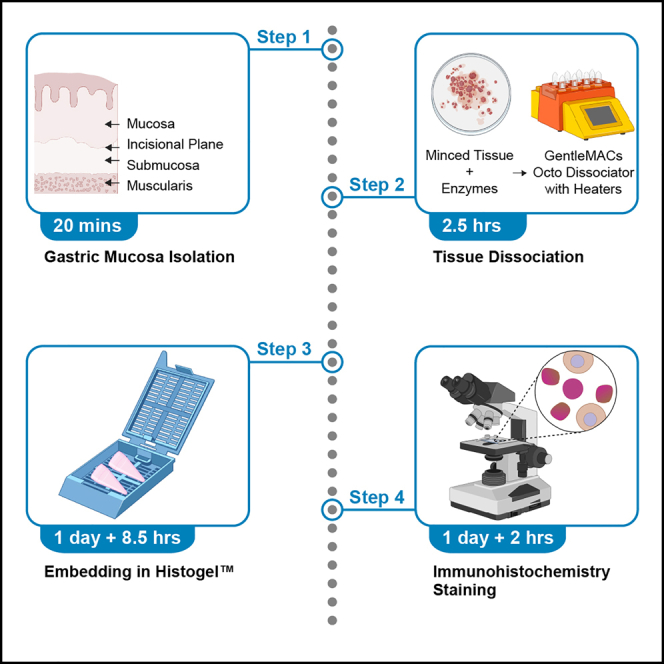

## Before you begin

The protocol outlined below describes the specific surgical and laboratory steps for isolating signet ring cells from human gastric tissue. However, this protocol may be applied to other species, and may be appropriate for isolating other cell types present in gastric mucosa.

### Institutional permissions

All experiments involving human samples must be approved by institutional permissions and national laws and regulations. All clinical protocols in this study were approved by the Institutional Review Board and informed consent was obtained from all research subjects. Additional permissions should be granted from institutions providing human tissues. Surgical experience is recommended for procurement of gastric mucosa.

### Prepare buffers and enzymes

**Timing: 40 min**

1. Ensure that all reagents are available in sufficient volume prior to beginning, especially 0.04% BSA in 1× PBS and staining reagents.

***Note:*** 0.04% BSA in 1× PBS should be prepared first before other reagents. BSA will take several minutes to dissolve. Please see the materials and equipment section for detailed preparation instructions.

***Note:*** Please see the materials and equipment section for detailed stain preparation and the procedure for testing solution viability prior to use.

2. Aliquot 10–20 mL of MACS Tissue Storage Solution into a 50 mL centrifuge tube and store on ice.
3. Thaw aliquoted Miltenyi Biotec Tumor Dissociation Kit, Human enzymes on ice.

***Note:*** Do not mix enzymes until tissue is ready for dissociation as doing so will prematurely activate enzymes.

## Key resources table

**Table:** 

REAGENT or RESOURCE	SOURCE	IDENTIFIER
**Antibodies**		

E-cadherin (24E10) rabbit mAb #3195 (1:400)	Cell Signaling Technology	Cat#3195; RRID: AB_2291471
Goat anti-rabbit IgG antibody (H + L), biotinylated (1:100)	Vector Labs	SKU BA-1000-1.5; RRID: AB_2313606

**Chemicals, peptides, and recombinant proteins**		

Fisher BioReagents bovine serum albumin (BSA) microbiological grade powder	Fisher	Cat#BP9700100
Thermo Scientific Chemicals phosphate-buffered saline (PBS, 1×), sterile-filtered	Fisher	Cat#AAJ61196AP
Gibco DMEM, no glucose	Fisher	Cat#11-966-025
Basic fuchsin hydrochloride, MP Biomedicals	Fisher	Cat#ICN15042305
Hydrochloric acid, 1 N standard solution, Thermo Scientific Chemicals	Fisher	Cat#AC124210025
Sodium hydrogen sulfate, anhydrous, 90+%, remainder mainly sodium sulfate, Thermo Scientific Chemicals	Fisher	Cat#AAB2558730
Periodic acid (white to pale-yellow crystals or white powder), Fisher BioReagents	Fisher	Cat#BP581-100
Lithium carbonate (powder/certified ACS), Fisher Chemical	Fisher	Cat#L119-500
Antigen unmasking solution, citrate-based	Vector Labs	Cat#H-3300-250
Tissue-Tek 10% neutral buffered	Sakura	Product code 5993
Tissue-Tek reagent alcohol 70%	Sakura	Product code 6021
IMEB, Inc reagent alcohol, 80%	Fisher	Cat#NC1897349
Tissue-Tek reagent alcohol 95%	Sakura	Product code 6020
Tissue-Tek reagent alcohol 100%	Sakura	Product code 5987
Tissue-Tek xylene	Sakura	Product code 5988
Tissue-Tek paraffin	Sakura	Product code 4005
Fisher Chemical Permount mounting medium	Fisher	Cat#SP15-100
Mercedes Medical glass coverslips	Fisher	Cat#15-183-95
Fisherbrand economy plain glass microscope slides	Fisher	Cat#12-550-A3
Gibco trypan blue solution, 0.4%	Thermo Fisher Scientific	Cat#15250061
MACS tissue storage solution	Miltenyi Biotec	Cat#130-100-008
Gibco TrypLE express enzyme (1×), no phenol red	Thermo Fisher Scientific	Cat#12604013
HistoGel specimen processing gel, Epredia	VWR	Cat#83009-992

**Critical commercial assays**		

Tumor dissociation kit, human	Miltenyi Biotec	Cat#130-095-929

**Other**		

VWR standard line sterile centrifuge tubes with flat caps, conical bottom, PP, 50 mL	VWR	Cat#10026-078
Corning disposable vacuum filter/storage systems	Fisher	Cat#09-761-100
Eisco wax-lined dissection tray	VWR	Cat#470324-790
Dissecting specimen pins, Mortech	VWR	Cat#76549-022
Sharp-point surgical scissors	VWR	Cat#470018-940
Feather single-use scalpels #15	Fisher	Cat#08-927-5C
Integra Miltex Gerald dressing forceps	Fisher	Cat#12-460-534
OHAUS Gold series pocket scales	Fisher	Cat#S93805
Corning not TC-treated culture dishes, 100 × 20 mm	Corning	Cat#430588
gentleMACS Octo dissociator with heaters	Miltenyi Biotec	Cat#130-096-427
gentleMACS C tubes	Miltenyi Biotec	Cat#130-093-237
MACS SmartStrainers (70 μm)	Miltenyi Biotec	Cat#130-098-462
VWR Mega Star 1.6R general purpose 1.6 L benchtop centrifuge, refrigerated, 120 V (centrifuge only)	VWR	Cat#76468-132
VWR Mega Star 1.6R general purpose 1.6 L benchtop centrifuge tissue culture package, refrigerated, 120 V	VWR	Cat#76519-280
Falcon cell strainers, sterile, Corning, 40 μm	VWR	Cat#352340
Nexcelom Cellometer Auto T4 Plus cell counter	Nexcelom Bioscience LLC	SKU: Cellometer Auto T4 Plus
VWR standard line sterile centrifuge tubes with flat caps, conical bottom, PP, 15 mL	VWR	Cat#10026-076
Eisco Chattaway lab spatula	Fisher	Cat#S27089
Fisherbrand cutting board	Fisher	Cat#09-002-24A
Fisherbrand razor blades	Fisher	Cat#12-640
Tissue-Tek processing/embedding cassette, tan; 1500/cs	Sakura	Product code 4125
Tissue-Tek VIP 6 AI vacuum filtration processor	Sakura	Product code 6040
Cytiva Whatman qualitative filter paper: grade 1 circles	Fisher	Cat#09-805P
PYREX watch glass/beaker cover with fire-polished edges	Fisher	Cat#02-212A

## Materials and equipment

• 0.04% BSA in 1× PBS: dissolve 2 g Bovine Serum Albumin in 50 mL 1× PBS, dilute 1:100 in 1× PBS.

**Table 1:** Schiff’s Leuco-Fuchsin solution

Reagent	Final concentration	Amount
Basic Fuchsin	0.45%	1 g
Distilled Water	90.01%	200 mL
1 N HCl	9.09%	20 mL
Anhydrous Sodium Bisulfite or Sodium Meta-Bisulfite	0.45%	1 g

***Note:*** All BSA solutions should be prepared at 4°C to limit reagent degradation and bacterial growth. Mitigate the production of inaccurate solutions by making a concentrated solution and diluting down. Start by making a solution of 4% BSA in 1× PBS by adding 2 g of Bovine Serum Albumin to 50 mL 1× PBS. Dissolve with a magnetic stir bar for several minutes. Dilute 1:10 first by adding 10 mL 4% solution to 40 mL 1× PBS. Dilute 1:10 again by adding 10 mL 0.4% solution to 40 mL 1× PBS to obtain a final concentration of 0.04% BSA in 1× PBS. Avoid creating bubbles in the solution during mixing, which may introduce contamination. Sterilize the 0.04% BSA solution via vacuum filtration system or syringe filter. Ensure a filter pore size of no greater than 0.22 μm for adequate sterility. To use the Corning Disposable Vacuum Filter/Storage System, pour 0.04% BSA into the funnel reservoir, connect the angled hose connector to a vacuum source, and filter until all solution is transferred to the receiver bottle.

***Note:*** Reconstituted BSA is stable at 4°C for 2–7 days, however rapid degradation is exacerbated by improper storage temperatures. It is recommended to prepare this solution fresh at 4°C for best results and to store on ice prior to use.

***Note:*** Add Basic Fuchsin to distilled water and bring solution to boiling point (Table 1). Cool to 50°C, filter with Cytiva Whatman Qualitative Filter Paper: Grade 1 Circles, then add HCl. Cool to room temperature of 20°C–22°C, then add Anhydrous Sodium Bisulfite. Protect from light for 48 h until solution becomes straw or white colored. Add a teaspoon (approximately 1 g) of activated charcoal accompanied by shaking and filtering to clear the reagent. A colorless solution is ready for use, otherwise charcoal and filtering steps must be repeated. The reagent is hazardous; please use personal protective equipment and care when in use. See Critical note below for details.

***Note:*** This solution expires in a week when stored properly at 4°C. Carefully examine Schiff’s Leuco-Fuchsin Solution prior to use. Colorless solution is acceptable, pink solution must be replaced. Additionally, test Schiff’s Solution before use by pouring a few drops into 10 mL of 37%–40% formaldehyde in a watch glass. A rapid reddish-purple color shift indicates the solution is acceptable for use. A delayed and deep blue-purple color shift indicates the solution has degraded and must be remade fresh.

**CRITICAL:** Schiff’s Leuco-Fuchsin Solution is an acute oral toxin and can cause serious skin and eye damage, corrosion, or irritation. It is a respiratory tract irritant and also an OSHA category 2 carcinogen suspected of causing cancer. Use personal protective equipment such as gloves, a lab coat, and eye protection. Prepare and use under a fume hood or in a well-ventilated area.

• 1% Periodic Acid Solution: add 1 g Periodic Acid Crystals in 100 mL distilled H_2_O.

***Note:*** Store at 4°C for up to a week.

**CRITICAL:** 1% Periodic Acid Solution is highly corrosive and causes severe skin and eye damage. Use personal protective equipment such as gloves, lab coat, and eye protection when preparing and handling. Prepare and use under a fume hood, in a well-ventilated area, or with a respirator.

• 1% Lithium Carbonate: add 1 g Lithium Carbonate in 100 mL distilled H_2_O.

***Note:*** Store at room temperature of 20°C–22°C in a well-ventilated place.

**CRITICAL:** 1% Lithium Carbonate is an OSHA category 2 mutagenic suspected of causing genetic defects and causes both skin and eye irritation. Use personal protective equipment such as gloves, lab coat, and eye protection when preparing and handling. Prepare and use under a fume hood, in a well-ventilated area, or with a respirator.

• 1× Antigen Unmasking Solution, Citrate-Based: Dilute 100× stock to 1× working stock with distilled water.

***Note:*** It may be helpful to dilute from 100× to 1× using the technique described above in the note on preparation for 0.04% BSA in 1× PBS.

***Note:*** Store at 4°C and keep container tightly closed. Store locked up in a well-ventilated area.

**CRITICAL:** 100× stock Antigen Unmasking Solution, Citrate-Based causes serious eye damage, and may cause respiratory irritation and organ damage through prolonged or repeated exposure. Wear appropriate gloves, eye protection, lab coat, and respirator if ventilation is poor.

***Alternatives:*** In this protocol we used the Nexcelom Cellometer Auto T4 Plus Cell Counter for counting cells, but other models and manual counting may be appropriate.

***Alternatives:*** Prior to embedding and staining, it is optimal to fix in 70% ethanol or 10% neutral buffered formalin (NBF) for 24–48 h. Alternatively, cells can be fixed in formalin for 24 h. If processing cannot occur immediately following the 24 h incubation period, the formalin must be carefully removed from the cells without disturbing the cell pellet and replaced with an equal volume of 70% ethanol while maintaining pellet integrity. Cells in ethanol can be stored indefinitely at 4°C until trimming.

***Alternatives:*** The recommended processor for embedding is the Sakura Tissue-Tek VIP 6 AI Vacuum Filtration Processor which dehydrates, clears, and infiltrates samples with liquid paraffin and can be utilized with embedded HistoGel samples. Similar instruments may be appropriate.

## Step-by-step method details

### Isolation of gastric mucosa

**Timing: 20 min**

This step separates the gastric mucosa, which includes epithelium and lamina propria layers, from the underlying submucosa and deeper muscle layers. Surgical experience is recommended.

**Figure 1:**
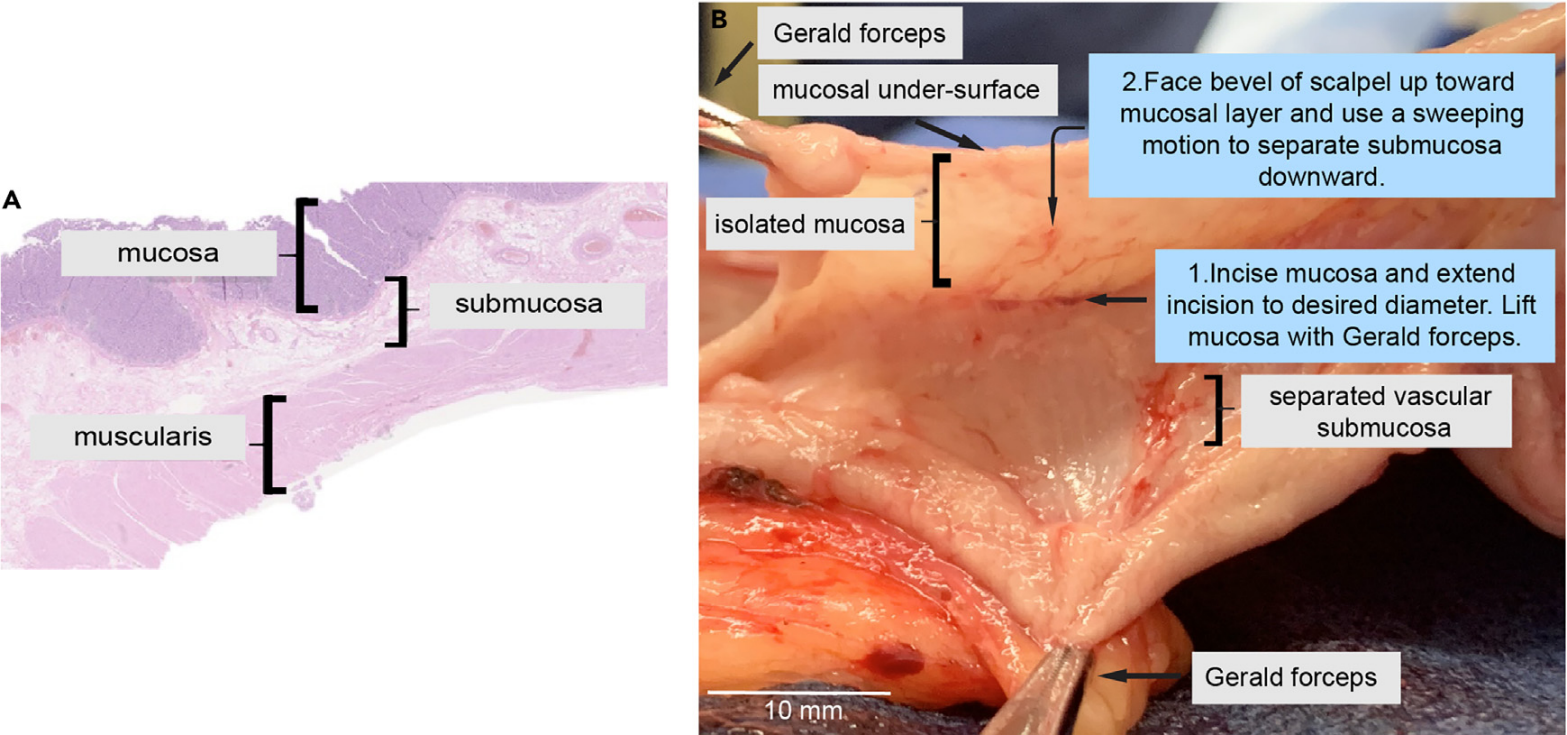
Isolation of gastric mucosa from submucosa during tissue procurement (A) A hematoxylin-eosin stained slide of gastric tissue demonstrates the layers of the stomach at 200× total magnification. (B) Submucosa is vascular and web-like in appearance. A #15 blade scalpel is used to incise the mucosa and to separate the submucosa from the undersurface of the mucosa in gentle 2–3 mm strokes.

***Note:*** A small 1–2 cm diameter piece of isolated mucosa is generally sufficient to obtain 0.2–1 g of tissue. We recommend procuring approximately 1 g of isolated mucosa for this protocol.

1. Remove the total gastrectomy specimen from the surgical field and place the specimen on a clean back table.
2. Remove the perigastric soft tissues with scissors.
3. Incise the stomach along the greater curvature. Perform steps 1a-e with an assistant.
  a. Expose the entire gastric mucosa of the stomach and pin the stomach to a cork or wax board with the mucosal surface face-up.
  b. Identify the mucosal region(s) of interest.
    ***Note:*** The mucosal area should be approximately 5 cm^2^.
  c. Gently incise the mucosa with a #15 scalpel and lift the mucosa using Gerald forceps.
    **CRITICAL:** Handle the tissue with care until the harvest is complete. The mucosal layer is thin and may tear easily when separating from the submucosa. Large tears or holes in the mucosa may result in having to restart the dissection.
    ***Note:*** Gerald forceps are recommended for their precise and gentle grip, which prevent slippage and tearing of the tissue.
  d. Extend the incision to the desired diameter at the interface of mucosal and submucosal layers.
  e. Gently lift the mucosal layer upward and secure the remainder of the tissue flat against the working surface.
    ***Note:*** Assistance with an additional Gerald forceps is recommended when gently lifting the mucosal layer upward, see Figure 1.
    ***Note:*** The submucosa is white and web-like in structure and contains small blood vessels that can aid in appropriately differentiating mucosal from submucosal layers. The tiny vessels should remain with the submucosa, and thus separated from the mucosal layer. Transillumination may also help. Meticulous separation of the gastric mucosa from submucosa is imperative for a high-yield single cell suspension.
  f. Use the scalpel with the bevel facing up toward the under-surface of the mucosa to gently brush the submucosa down and simultaneously lift the mucosal layer upward.
4. Mass the isolated mucosa in a dry petri dish immediately using a digital pocket scale and continue procurement as needed to obtain ≥1 g of tissue.

5. Record the mass of isolated mucosa and immediately transfer isolated mucosa to MACS Tissue Storage Solution for transport on ice.

### Dissociation of gastric mucosa

**Timing: 2.5 h**

In this step, dissociate the gastric mucosa into a single cell suspension.^1^ This is required for downstream applications, such as scRNA-seq and flow cytometry.^2^^,^^3^

6. Transfer isolated mucosa to a petri dish with forceps.

***Note:*** Limit transfer of MACS® Tissue Storage Solution into petri dish.

**Figure 2:**
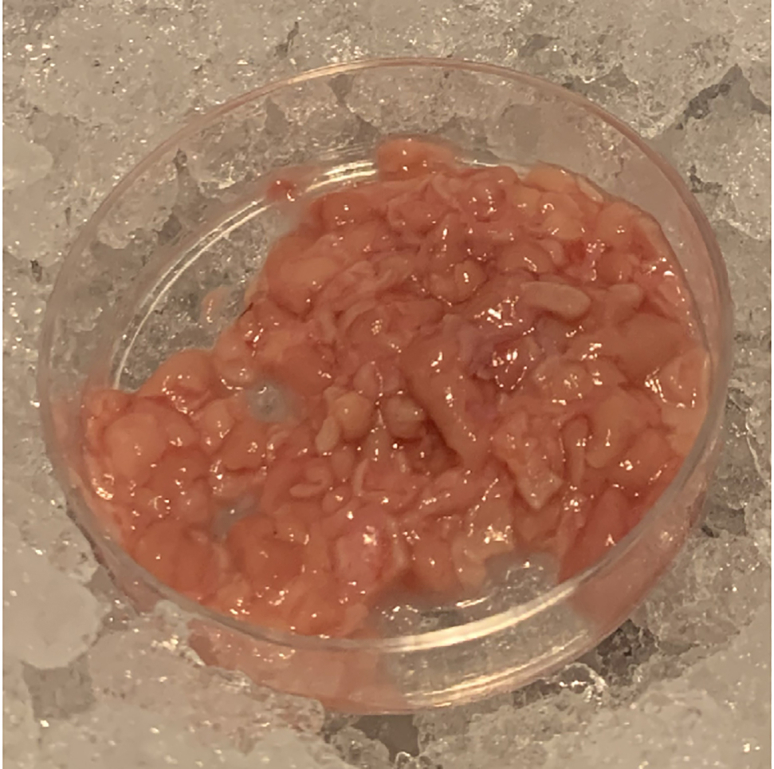
Minced gastric mucosa during dissociation Procured gastric mucosa should be minced into 1–2 mm pieces. Gastric mucosa should be kept on ice during mincing to slow molecular degradation.

***Note:*** Excessive mincing with surgical scissors or scalpel may induce cell death and low viability. Keep dish on ice while mincing to limit cell death.

7. Mince 0.2–1 g isolated mucosa with scissors or scalpel into 1–2 mm pieces, see Figure 2.

8. Begin Miltenyi Biotec Tumor Dissociation Kit, Human protocol as follows:
  a. Add 4.7 mL 4°C DMEM, 200 μL Enzyme H, 100 μL Enzyme R, and 25 μL Enzyme A to a gentleMACS C Tube.
  b. Add minced tissue to gentleMACS C Tube.
  c. Slide the heater onto the gentleMACS C Tube and secure to the gentleMACS Octo dissociator.
  d. Select the pre-programmed protocol “37C_h_TDK_1 for soft tissue” dissociation and start run.
9. Filter cell solution as follows:
  a. Filter cell solution through a 70 μm cell strainer into a 50 mL conical tube.
    ***Note:*** If filtering is slow due to a viscous cell solution, resuspend the cell solution in 5 mL added DMEM and centrifuge at 400 × *g* for 5 min at 4°C. This technique can be repeated at any point during experimentation to reduce viscosity. Do not use a plunger to push a viscous cell suspension through the cell strainer. Doing so may reduce the viability of the final cell product.
  b. Wash the gentleMACS C Tube and cell strainer with 5 mL 4°C DMEM. Centrifuge cell solution at 400 × *g* for 5 min at 4°C.
  c. Repeat filtering and washing steps with a 40 μm cell strainer.
  d. Carefully decant and pipette off media supernatant.
  e. Resuspend pellet and wash with 8 mL 0.04% BSA in 1× PBS. Centrifuge the cell solution at 400 × *g* for 5 min at 4°C.
    ***Optional:*** If the cell pellet is particularly sticky and the cells will not resuspend easily as single cells, consider resuspending pellet in 2–3 mL of 1× TrypLE and gently agitate for no more than 2 min. Follow by quenching TrypLE with 20 mL 0.04% BSA in 1× PBS and centrifuging at 400 × *g* for 5 min at 4°C.^4^
  f. Resuspend cells in 1 mL 0.04% BSA in 1× PBS and dilute 1:1 with Trypan Blue for cell counting.
    ***Note:*** If cell viability assessment is not required for downstream applications, this step may be omitted.
    ***Note:*** Complete additional washes with 0.04% BSA in 1× PBS for future applications that are sensitive to EDTA.
    ***Note:*** In this protocol we used the Nexcelom Cellometer Auto T4 Plus Cell Counter, but other models and manual counting may be used.

### Embedding in HistoGel for immunohistochemistry staining

**Timing: 1 day + 8.5 h**

In this step, fix and embed the dissociated cells in a solid medium. We used HistoGel Specimen Processing Gel to preserve the histologic and cytologic features of the dissociated single cell gastric mucosa prior to immunohistochemical analysis.^5^^,^^6^

10. Fix cells optimally in 70% ethanol or 10% neutral buffered formalin (NBF) for 24–48 h in a 15 mL conical tube.

**Figure 3:**
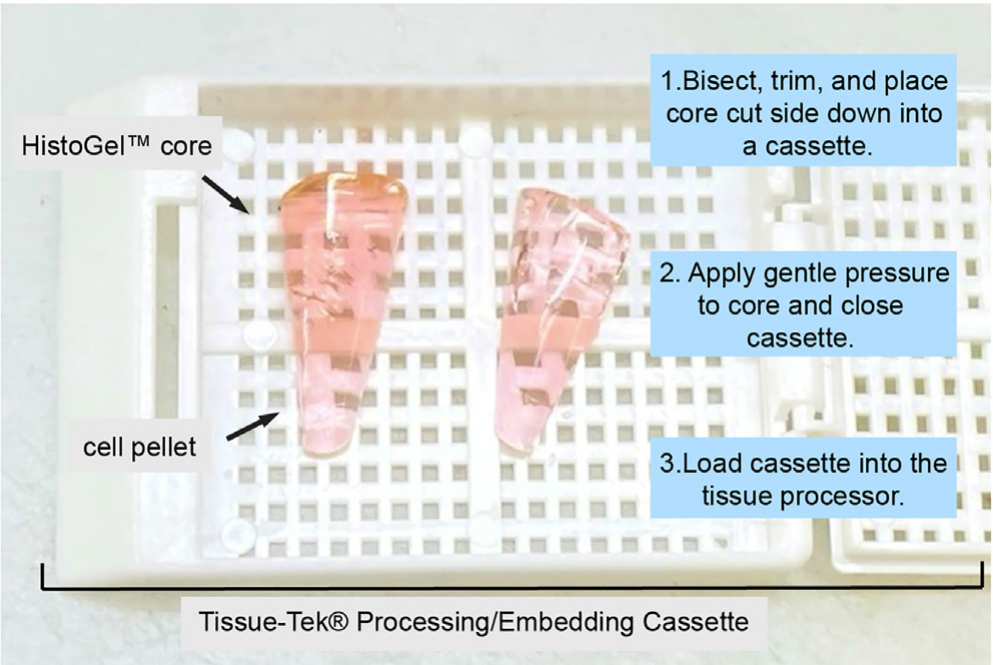
Embedding cells in HistoGel for immunohistochemistry staining The solidified HistoGel core containing cells should be bisected into two equally sized halves and placed cut side down into a Tissue-Tek Processing/Embedding Cassette. Gentle pressure should be applied to the core to ensure that all cells are embedded into the same plane. The cassette is then closed and loaded into the Tissue-Tek VIP 6 AI Vacuum Filtration Processor for tissue processing.

**Pause point:** Cells can be stored indefinitely at 4°C or until trimming.

11. Perform HistoGel trimming as follows:
  a. pellet cells at 1000 × *g* for 5 min.
  b. Remove ethanol or formalin supernatant. Do not disturb cell pellet.
  c. Add 1 mL preheated and liquefied HistoGel into the 15 mL conical tube containing cells.
  d. Gently tap the bottom of the tube to dislodge cell pellet from the bottom of the tube. Ensure that the HistoGel completely surrounds the pellet before solidifying.
  e. Solidify the HistoGel by placing the tube on ice for approximately 2 min.
  f. Remove the HistoGel core containing cells from the 15 mL tube.
    ***Note:*** A laboratory spatula may be used to gently pry the core from the sides and bottom of the tube. Be sure not to damage the cell pellet encapsulated in the core during removal.
    **Pause point:** Cells embedded in HistoGel may be stored indefinitely before staining.
  g. Place the HistoGel core on a cutting board.
  h. Cut the core longitudinally through the center with a razor blade, bisecting the core into two evenly sized halves.
  i. Trim excess HistoGel away from the core.
    ***Note:*** This should be gel that was closest to the top of the 15 mL tube, which does not contain any cells.
  j. Place both trimmed halves cut side down into a pre-labeled Tissue-Tek Processing/Embedding Cassette and apply gentle pressure to ensure all cells are embedded in the same plane, see Figure 3.
  k. Load Tissue-Tek Processing/Embedding Cassette into the Tissue-Tek VIP 6 AI Vacuum Filtration Processor and utilize the tissue processing protocol outlined below (Table 2).

**Table 2:** Tissue processing protocol

Steps	Reagent	Time (min)	Temperature (°C)
1	70% Ethanol	30	RT
2–3	80% Ethanol	30	RT
4–5	95% Ethanol	30	RT
6–8	100% Ethanol	30	RT
9–10	100% Xylenes	30	RT
11–13	Paraffin	45	60
14	Paraffin	30	60

### Immunohistochemistry staining

**Timing: 1 day + 2 h**

In the absence of specific signet ring cell antibodies, PAS and E-Cadherin double staining can be used to visualize suspected signet ring cells.^7^^,^^8^^,^^9^^,^^10^^,^^11^^,^^12^

12. Perform PAS staining as follows:
  a. Deparaffinize and hydrate slides to water.
  b. Immerse in 1% Periodic Acid Solution for 8 min and several times with distilled water.
  c. Immerse in Schiff’s Leuco-Fuchsin Solution for 10 min and wash in running tap water for 5 min.
  d. Counterstain in Mayer’s hematoxylin for 15 s and wash in running tap water.
  e. Dip slide into 1% Lithium Carbonate or other suitable bluing reagent for 1–2 s and wash in running tap water for 5 min.
  f. Dehydrate and clear in xylene.

**Pause point:** Double staining procedure can be paused indefinitely at this step.

13. Perform E-cadherin staining as follows:
  a. Complete antigen retrieval using 1× Antigen Unmasking Solution, Citrate-Based (Vector Labs Cat# H-3300-250) for 10 min at 100°C.
  b. Begin E-cadherin staining by diluting E-Cadherin primary antibody 1:400 (Cell Signaling Technologies Cat #3195, RRID: AB_2291471).
  c. Incubate in primary antibody for approximately 16 h at 4°C according to the manufacturer’s staining protocol www.cellsignal.com/products/primary-antibodies/e-cadherin-24e10-rabbit-mab/3195.
  d. Dilute Biotinylated Goat anti-Rabbit (Vector Labs SKU BA-1000-1.5, RRID: AB_2313606) 1:100 for use in secondary antibody staining.
  e. Mount with Permount and a cover slip.

## Expected outcomes

**Figure 4:**
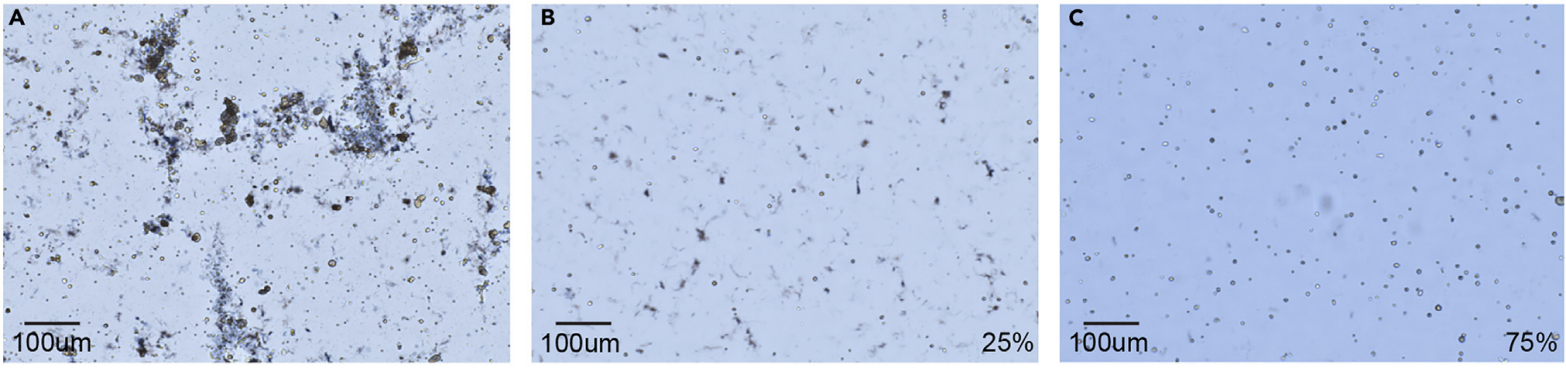
Viability of epithelial cells is sensitive to gastric mucosa isolation technique and washes (A) Poorly executed isolation technique results in clumpy, inadequately dissociated suspensions with large and abundant cellular debris. Viability assessment of such low quality suspensions is not possible. (B) Meticulous isolation technique produces significantly less cellular debris than (A), however small debris misidentified as dead cells artificially lower cell viability to 25%. (C) An additional 2 washes for the same sample as (B) with 0.04% BSA in 1× PBS removes significant debris, improving viability to 75%. All images are cropped and captures by Nexcelom Cellometer Auto T4 Plus cell counter at 4× total magnification.

**Figure 5:**
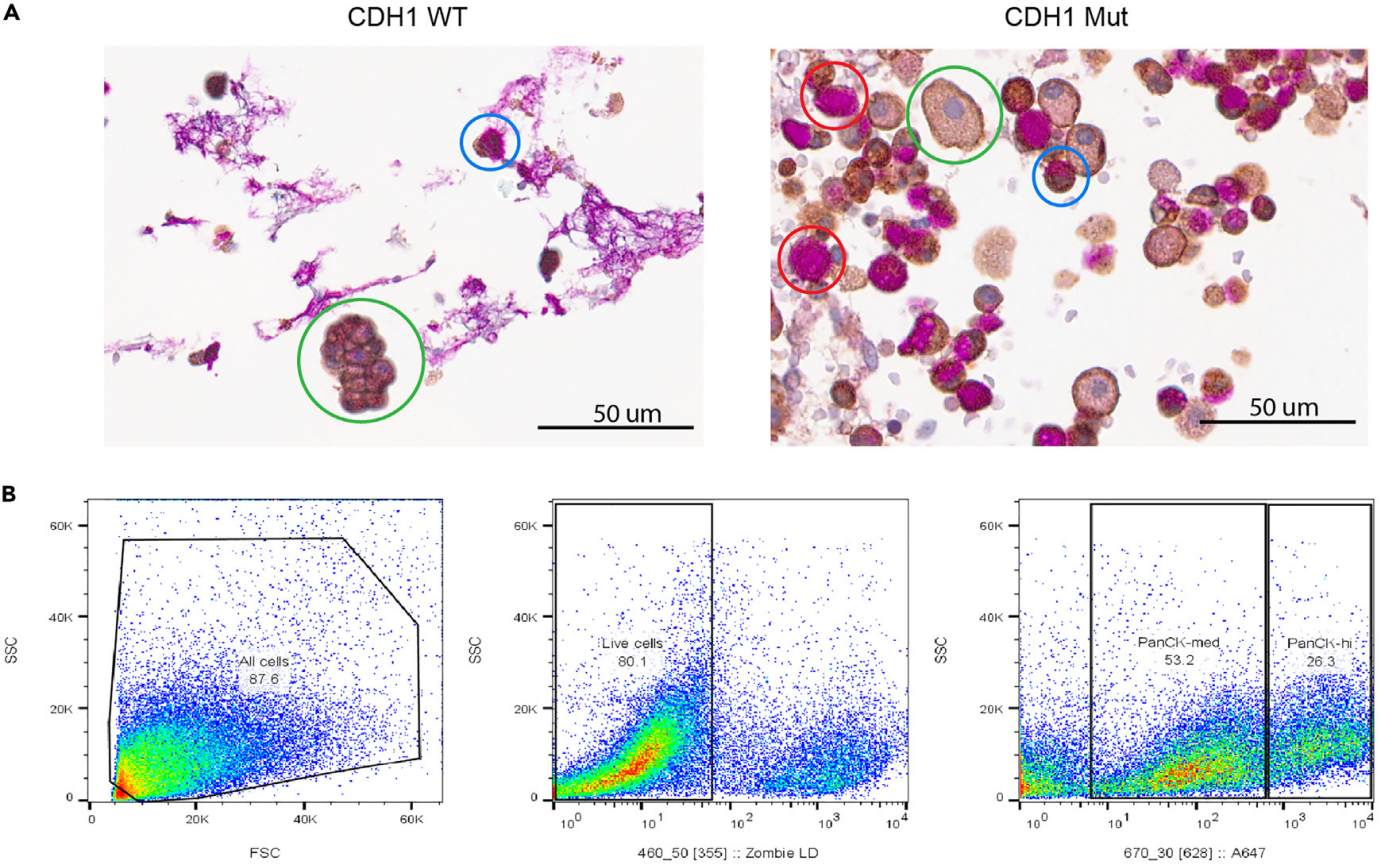
Downstream applications of isolated gastric mucosa (A) Dissociated gastric mucosa in HistoGel medium prepared for periodic acid-Schiff and E-cadherin double stain immunohistochemistry for a patient with CDH1 mutation and a wild-type negative control at 400× total magnification. Parietal cells are marked in blue, chief cells in green, and signet ring cells in red based on staining and morphology. Cells expressing E-cadherin are stained brown. Early signet ring cell lesions are E-cadherin negative and PAS positive. (B) Flow cytometry scatterplot of a single cell suspension of CDH1 gastric mucosa demonstrates a viable sample. 80.1% viability with Zombie Live-Dead staining (BioLegend Cat#423105) with significant cytokeratin expression via Alexa Fluor 647-conjugated pan-cytokeratin monoclonal antibody (BioLegend Cat #628604), as seen in gastric epithelium and signet ring cells.

Approximate yield is 5–10 million live cells per gram of gastric mucosa using an automated cell counter; however, both yield and viability are critically dependent on efficient processing during gastric mucosa isolation and dissociation. In our experiments, we typically obtain >75% cell viability, see Figures 4 and 5.

This protocol for single cell dissociation in gastric mucosa tissue can be utilized for downstream techniques such as immunohistochemistry, flow cytometry, and scRNA-seq to interrogate gastric cancer biology, for example signet ring cell carcinoma, see Figure 5. This protocol produces reliable high-yield and high-viability single cell suspensions of gastric mucosa and may be adapted to examine other cell types.

## Limitations

Our proposed single cell dissociation protocol has several limitations, most of which occur at the time of tissue procurement and may impact dissociation efficiency and cell viability. Epithelial cells of the gastric mucosa are inherently difficult to dissociate due to both tight and adherens junctions.^13^ Extracellular matrix proteins, which are abundant in the submucosa, make filtering and enzymatic dissociation challenging if submucosa is present in the isolated sample. Therefore, isolating the gastric mucosa to exclude submucosa at tissue procurement is a critical step. Successful isolation technique relies heavily on surgical expertise. Prolonged isolation and laboratory steps delayed by troubleshooting efforts decrease cell viability. Performing a trial run is recommended prior to protocol use on limited patient tissue.

## Troubleshooting

### Problem 1

Difficulty identifying mucosa and submucosa during isolation of gastric mucosa.

### Potential solution

Several structural differences exist that differentiate mucosa and submucosa. The submucosal layer is very thin, web-like, contains small blood vessels, and is white in color. The mucosal layer is thicker and light pink in color. Transillumination may help to visualize these differences.

### Problem 2

Tissue tearing during isolation of gastric mucosa.

### Potential solution

Several gentle 2–3 mm strokes with a #15 blade scalpel are more effective in isolating the mucosa than larger harsh strokes which can cause the tissue to tear. Use Gerald forceps to grip the tissue and prevent slippage and tears. Use an assistant.

### Problem 3

Clogged cell strainers during dissociation of gastric mucosa.

### Potential solution

There are many reasons why the cell strainer may clog during dissociation. We provide a list of solutions.

• Insufficient isolation of the gastric mucosa from the submucosa at the time of tissue procurement contributes to a viscous “slimy” texture of digested gastric mucosa, which easily clogs filters. Ensuring that extreme care is taken during tissue procurement to remove as much submucosa as possible.
• Filtering first through a 70 μm cell strainer before proceeding to a 40 μm cell strainer may also help.
• Diluting digested tissue first with 5 mL 4°C DMEM prior to the first filtering step. Centrifuge solution at 400 × *g* for 5 min at 4°C.

### Problem 4

Low cell viability after dissociation of gastric mucosa.

### Potential solution

Cell death begins once the stomach is removed from the patient. To avoid low cell viability.

• Ensure the tissue is processed at 4°C and with reagents cooled to 4°C from procurement until the end of dissociation.
• Excessive mincing is not necessary and may induce unwanted cell death. All mincing must be performed in a petri dish on ice.

### Problem 5

Cell clumping in single cell suspension during dissociation of gastric mucosa.

### Potential solution

Increase the BSA concentration of the 0.04% BSA in 1× PBS solution and consider titrating the optional TrypLE step with different volumes of TrypLE (ex: 1, 2, 3, 4 mL) and incubation times (ex: 30, 60, 90, 120 s).

## Resource availability

### Lead contact

Further information and requests for resources and reagents should be directed to and will be fulfilled by the lead contact, Jeremy L. Davis, MD (jeremy.davis@nih.gov).

### Materials availability

This study did not generate unique reagents.

### Data and code availability

This study did not generate/analyze datasets/code.
